# Heralded quantum repeater based on the scattering of photons off single emitters using parametric down-conversion source

**DOI:** 10.1038/srep28744

**Published:** 2016-06-28

**Authors:** Guo-Zhu Song, Fang-Zhou Wu, Mei Zhang, Guo-Jian Yang

**Affiliations:** 1 Department of Physics, Applied Optics Beijing Area Major Laboratory, Beijing Normal University, Beijing 100875, China

## Abstract

Quantum repeater is the key element in quantum communication and quantum information processing. Here, we investigate the possibility of achieving a heralded quantum repeater based on the scattering of photons off single emitters in one-dimensional waveguides. We design the compact quantum circuits for nonlocal entanglement generation, entanglement swapping, and entanglement purification, and discuss the feasibility of our protocols with current experimental technology. In our scheme, we use a parametric down-conversion source instead of ideal single-photon sources to realize the heralded quantum repeater. Moreover, our protocols can turn faulty events into the detection of photon polarization, and the fidelity can reach 100% in principle. Our scheme is attractive and scalable, since it can be realized with artificial solid-state quantum systems. With developed experimental technique on controlling emitter-waveguide systems, the repeater may be very useful in long-distance quantum communication.

The realization of long-distance entanglement plays an important role in quantum communication, such as quantum key distribution^1^,^2^,^3^, quantum dense coding^4^,^5^, quantum teleportation^6^, quantum secret sharing^7^, quantum secure direct communication^8^,^9^,^10^,^11^, and so on. However, due to the thermal fluctuation, vibrations, and other imperfections, inevitable exponential scaling errors occur on the quantum state of photons with the transmission distance in the noisy channel. In order to construct a long-distance entangled channel, the concept of quantum repeater was originally proposed by Briegel *et al*.^12^ in 1998. Its basic idea is to divide the total transmission distance into several segments, and then use entanglement purification and entanglement swapping to suppress the influence of environment noises. In 2001, Duan *et al*.^13^ presented a proposal for quantum repeaters with atomic ensembles as quantum memories, known as the DLCZ protocol. In 2006, using only two qubits at each station, Childress *et al*.^14^ constructed a fault-tolerant quantum repeater, which provides the possibility to realize repeaters in simple physical systems such as solid-state single-photon emitters. In 2007, with two-photon Hong-Ou-Mandel-type interference, Zhao *et al*.^15^ proposed a robust and feasible quantum repeater. Meanwhile, Jiang, Taylor, and Lukin^16^ also put forward a robust scheme to construct a quantum repeater with atomic ensembles. In 2012, assisted by the spatial entanglement of photons and quantum-dot spins in optical microcavities, Wang, Song, and Long^17^ presented an efficient scheme for robust quantum repeaters. In 2014, Wang *et al*.^18^ proposed a scheme for a quantum repeater based on a quantum dot in an optical microcavity system. In 2015, Li and Deng^19^ presented a heralded high-efficiency quantum repeater with atomic ensembles and faithful single-photon transmission. Recently, Li, Yang, and Deng^20^ introduced another heralded quantum repeater for quantum communication network based on quantum dots embedded in optical microcavities, resorting to effective time-bin encoding. Furthermore, many experiments have been reported for building quantum repeaters, and remarkable progress has been made^21^,^22^,^23^,^24^,^25^,^26^,^27^.

In recent years, the scattering of photons off single emitters in one-dimensional (1D) waveguides has attracted much attention^28^,^29^,^30^,^31^,^32^,^33^,^34^,^35^,^36^,^37^,^38^,^39^. Single emitters can strongly interact with electromagnetic modes, and the scattering of photons off single emitters has been extensively explored. By employing various schemes with two- or three-level atoms, one can well control the propagation of single photon in 1D waveguides, and the quantum gates for quantum information processing have been realized^40^,^41^,^42^. In 2005, Shen and Fan^28^ discussed the interesting transport properties of a single photon interfering with the two-level emitters coupled to the modes in 1D waveguides. In 2007, Chang *et al*.^40^ implemented a single-photon transistor using nanoscale surface plasmons, in which strong nonlinear interactions between nanowires and waveguides are realized. In 2010, Witthaut and Sørensen^32^ solved the scattering problem for a single photon in a 1D waveguide coupled to a three-level emitter, and observed electromagnetically induced transparency for a driven Λ-system and V-system if both transitions couple to the waveguides. In 2012, based on the scattering of photons off single emitters in 1D waveguides, Li *et al*.^43^ presented an interesting scheme for realizing the robust-fidelity atom-photon entangling gate, in which the faulty events between photons and atoms can be turned into heralded losses.

In this paper, we exploit the scattering of photons off single emitters in 1D waveguides to construct a heralded quantum repeater, including robust nonlocal entanglement creation, entanglement swapping, and entanglement purification modules. Although great progress has been made, it is still a big challenge to obtain a long storage time in realistic quantum systems. In our scheme, we use a parametric down-conversion (PDC) source to create entangled photon pairs under the consideration that PDC sources are easily available with compact setups. Since atoms can provide coherence times as long as seconds, we choose a four-level atom as the emitter. It’s worth pointing out that, in our protocols, the faulty events can be turned into the detection of photon polarization, which can be immediately discarded. That is, the quantum repeater either succeeds with perfect fidelity or fails in a heralded way, which is very important for realistic quantum communication. With the remarkable progress on manipulating waveguide QED systems, there is no major difficulty to realize our scheme, and maybe it will have good applications in realistic long-distance quantum communication in future.

## Results

### The scattering of photons off single emitters in a 1D waveguide

**Figure 1:**
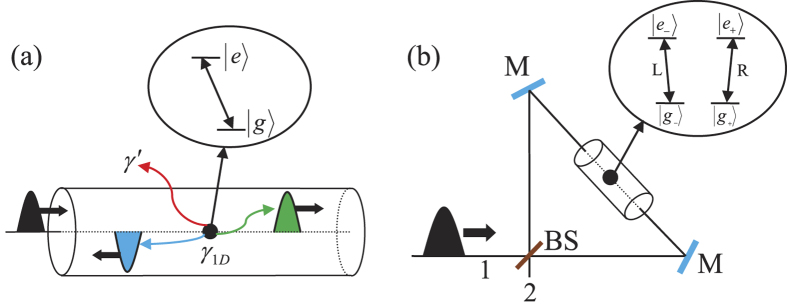
(**a**) The basic structure of a two-level atom (the black dot) embedded in a 1D waveguide (the cylinder). The atom acts as a photon mirror^28^, with its two levels |g〉 and |*e*〉 coupled via the waveguide. Under ideal resonance condition, an incident photon (black wave packet) is fully reflected (blue wave packet), or goes freely through (green wave packet) on the condition of detuning. (**b**) The heralded protocol for a robust-fidelity *Z* gate on an atom in a 1D waveguide. In fact, the emitter is a four-level atom, with degenerate ground states |g_±_〉 and degenerate excited states |*e*_±_〉. *BS* is a 50:50 beam splitter, M is a fully reflected mirror, and the black lines denote the paths of the travelling photon.

As illustrated in Fig. 1(a), the quantum system we consider is composed of a single emitter coupled to a 1D waveguide via electromagnetic interactions. The emitter is actually a simple two-level atom with the frequency difference *ω*_*a*_ between the ground state |g〉 and the excited state |*e*〉, and coupled to a set of traveling electromagnetic modes of the 1D waveguide. Under the Jaynes-Cummings model, the Hamiltonian for the system is^28^,^40^


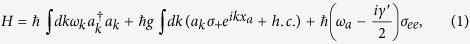


where *x*_*a*_ is the position of the atom, *a*_*k*_ (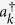) is the annihilation (creation) operator of the mode with the frequency *ω*_*k*_ (*ω*_*k*_ = *c*|*k*|, *k* is the wave vector), *σ*_+_ (*σ*_−_) is the atomic raising (lowering) operator, and *σ*_*ee*_ = |*e*〉〈*e*|. *γ*′ is the decay rate of the atom out of the waveguide, and *g* is the coupling strength between the atom and the electromagnetic modes of the 1D waveguide, assumed to be same for all modes.

Here, we focus on the scattering of a single photon, as shown in Fig. 1(a). By solving the scattering eigenvalue equation of the system (see the Methods section), one can obtain the reflection coefficient for the incident photon^40^


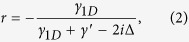


where *γ*_1*D*_ = 4*πg*^2^/*c* is the decay rate of the atom into the waveguide, and Δ = *ω*_*k*_ − *ω*_*a*_ is the frequency detuning between the photon and the atom. The transmission coefficient is given by *t* = 1 + *r*.

From Eq. (2), one can conclude that when the input photon resonates with the emitter (i.e., Δ = 0), the reflection coefficient changes into *r* = −1/(1 + 1/*P*), where *P* = *γ*_1*D*_/*γ*′ is the Purcell factor. As the spontaneous emission rate *γ*_1*D*_ into the 1D waveguide can be much larger than the emission rate *γ*′ into all other possible channels in a realistic atom-waveguide system^28^,^40^, one can get the reflection coefficient *r* ≈ −1. Therefore, for a large Purcell factor, the atom in state |g〉 acts as a nearly perfect mirror, which puts a *π*-phase shift on the reflected photon. Whereas, when the photon is decoupled from the two-level atom, nothing happens to the photon after the scattering process.

Now, we consider a four-level atom as the emitter in the 1D waveguide, as shown in Fig. 1(b). The atom has two degenerate ground states |*g*_±_〉 and two degenerate excited states |*e*_±_〉. The transition |g_−_〉 ↔ |*e*_−_〉 (|g_+_〉 ↔ |*e*_+_〉) is coupled to the *L*-polarized (*R*-polarized) photon, where *L* (*R*) denotes the left-circular (right-circular) polarization along the waveguide. Provided that the spatial wave function of the input photon from left is in the state |*ψ*〉, after the photon scatters with the atom, one gets the transformations as follows^43^:


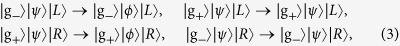


where |*ϕ*〉 = |*ϕ*_*t*_〉 + |*ϕ*_*r*_〉 represents the spatial state of the photon component left in the waveguide after the scattering process. Here, the states |*ϕ*_*t*_〉 = *t*|*ψ*〉 and |*ϕ*_*r*_〉 = *r*|*ψ*〉 denote the transmitted and reflected parts of the photon, respectively. If the incident photon is in the horizontal linear-polarization state 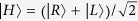, the corresponding transformations change into^43^


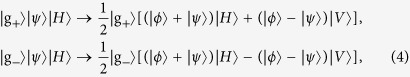


where 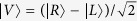 is the vertical linear-polarization state. It is meaningful that the scattering process generates a vertical-polarized component.

With the transformations discussed above, Li *et al*.^43^ presented a simple scheme for implementing a high-fidelity *Z* gate on an atom, as shown in Fig. 1(b). In detail, the incident photon in state |*H*〉 or |*V*〉 (from port 1) is first split by a 50 : 50 beam splitter (*BS*). The transmitted and reflected components scatter with the atom and exit the beam splitter simultaneously. Note that, due to quantum destructive interference, the two parts exit the beam splitter in port 1, without any photon component coming out from port 2. Finally, one obtains a high-fidelity atomic *Z* gate (marked by *Z*_*a*_) as follows:


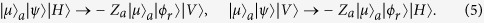


Here |*ϕ*_*r*_〉 = (|*ϕ*〉 − |*ψ*〉)/2 refers to the reflected part of the incident photon, and |*μ*〉_*a*_ is an arbitrary atomic superposition state in the basis {|0〉_*a*_ = |g_−_〉, |1〉_*a*_ = |g_+_〉}. The perfect scattering process occurs with the condition *P* → ∞, and we can get |*ϕ*_*r*_〉 = −|*ψ*〉. While for the imperfect situation with a finite *P*, |*ϕ*_*r*_〉 ≠ −|*ψ*〉, and the detection of an incorrectly polarized output heralds the failure of the corresponding gate. That is, the protocol for atomic *Z* gate works in a heralded manner.

### Robust entanglement creation for nonlocal atomic systems using a PDC source against collective noise

**Figure 2:**
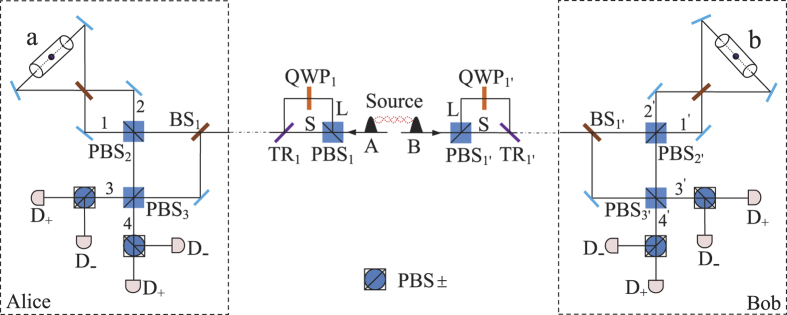
Schematic diagram for compactly implementing entanglement creation. *a* and *b* stand for two nonlocal atoms in 1D waveguides owned by Alice and Bob, respectively. *PBS*_*i*_ (*i* = 1, 2, 3, 1′, 2′, 3′) is a polarizing beam splitter which transmits the horizontal polarized photon |*H*〉 and reflects the vertical polarized photon |*V*〉. *QWP*_*i*_ (*i* = 1, 1′) is a quarter-wave plate to implement the conversion of the photon polarization. *PBS*± transmits photons with polarization |+〉 and reflects photons with polarization |−〉, where 
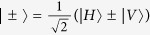
. *TR*_*i*_ (*i* = 1, 1′) is an optical device which can be controlled exactly as needed to transmit or reflect a photon, *BS*_*i*_ (*i* = 1, 1′) is a 50:50 beam splitter, and *D*_*i*_ (*i* = +, −) is a single-photon detector.

Now, let us describe the principle of our scheme for entanglement creation between two nonlocal atoms, as shown in Fig. 2. Here, two remote atom-photon subsystems are connected by a noisy quantum channel with a PDC source positioned at the middle point. Initially, in each subsystem, a stationary atom in the 1D waveguide, which is named as *a* (*b*) on the left (right) part of the setup, is prepared in the superposition state 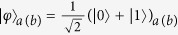, and a pair of photons A and B produced by the PDC source is in a common entangled state 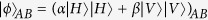, where 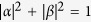. The state of the atom-photon system is


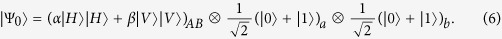


Our scheme for nonlocal entanglement creation works with the following steps.

First, the two entangled photons travel along the noisy channels in opposite directions. Each goes through a polarizing beam splitter (*PBS*) which transmits the photon component in state |*H*〉 and reflects the photon component in state |*V*〉. In detail, the photon A (B) in state |*H*〉 transmits through *PBS*_1_ (*PBS*_1′_), *TR*_1_ (*TR*_1′_) and goes directly into the noisy channel through the short path (S), while the photon A (B) in state |*V*〉 is reflected by the *PBS*_1_ (*PBS*_1′_) and passes through the quarter-wave plate *QWP*_1_ (*QWP*_1′_) to rotate its polarization. After the operation, the photon A (B) in the long path (L) is reflected by *TR*_1_ (*TR*_1′_) into the same noisy channel, but a little later than its early counterpart. *TR*_*i*_ (*i* = 1, 1′) is an optical device which can be controlled exactly as needed to transmit or reflect a photon. The state of the whole system at the entrance of the noisy channels changes into


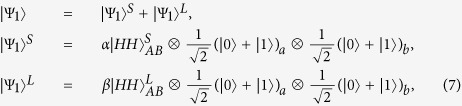


where 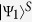 or 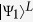 represents the state in which a photon travels along the short (S) or long (L) path. Henceforth, a state with superscript S or L follows the same regulation.

Second, the photons A and B, including their early component 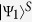 and late component 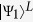, are transmitted to Alice and Bob via different noisy channels, respectively. Since the polarization states of the two components in photon A (B) are both |*H*〉, the influences of the collective noise in the noisy channel on them are the same ones^44^,^45^,^46^,^47^,^48^, which can be described by


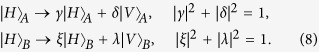


After the photons A and B travel in the corresponding quantum channels, the state of the whole system at the output ports of the channels evolves into |Ψ_2_〉, where


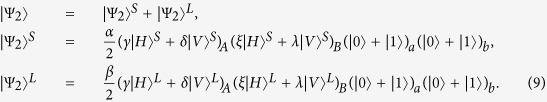


Third, getting out of the quantum channel, the early part and late part of photon A (B) travel through *BS*_1_ (*BS*_1′_). Since the late part |Ψ_2_〉^*L*^ undergoes the same processes as the early part |Ψ_2_〉^*S*^, to simplify the discussion, we just discuss the evolution of the early part in the following section. After passing through *BS*_1_ (*BS*_1′_), the transmitted component of early part travels to *PBS*_2_ (*PBS*_2′_), while the reflected component goes to *PBS*_3_ (*PBS*_3′_). After that, the state of the whole system evolves into |Ψ_3_〉, where


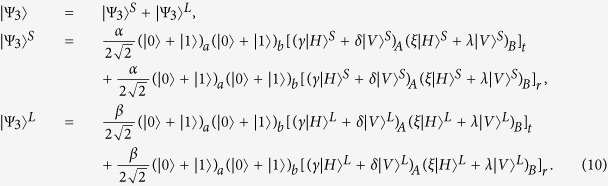


Here, the subscript *t* (*r*) represents the transmitted (reflected) component of photons. Subsequently, the transmitted component of photon A (B) passes through *PBS*_2_ (*PBS*_2′_), which transmits the photon in state |*H*〉 and reflects the photon in state |*V*〉. The component in state |*H*〉 of photon A (B) interacts with atom *a* (*b*) and exits the scattering setup in state |*V*〉 to *PBS*_2_ (*PBS*_2′_) in spatial mode 1 (1′), while the component in state |*V*〉 of photon A (B) also interacts with atom *a* (*b*) and exits the scattering setup in state |*H*〉 to *PBS*_2_ (*PBS*_2′_) in spatial mode 2 (2′). After above processes, the state of the whole system is changed from |Ψ_3_〉 to |Ψ_4_〉. Here,


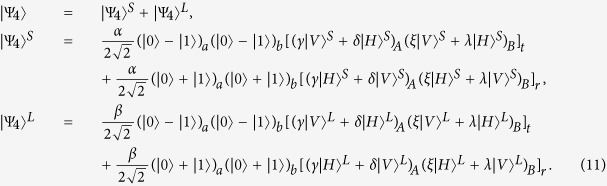


Fourth, the reflected part and the transmitted part of photon A (B) are rejoined in *PBS*_3_ (*PBS*_3′_). Then, the photon A (B) is separated into two parts: one goes into path 3 (3′) and the other one goes into path 4 (4′). The same process occurs to the part |Ψ_4_〉^*L*^ in a late time. The state of the whole system evolves into


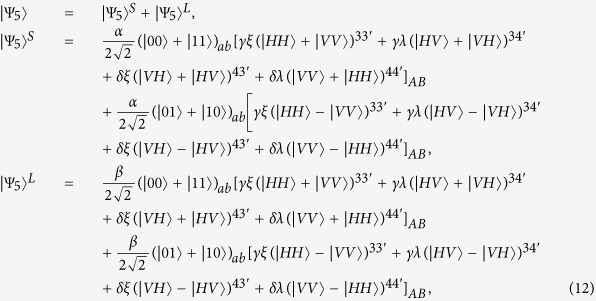


where the superscript *ij* (*i* = 3, 4 and *j* = 3′, 4′) indicates that photon A travels along path *i* and photon B along path *j* respectively. For example, 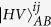 is in this case where photon A in state |*H*〉 travels along path *i* and photon B in state |*V*〉 along path *j*.

Fifth, the photons in paths 3 and 4 (3′ and 4′) both pass through a *PBS*±, and are detected by single-photon detectors *D*_+_ and *D*_−_ in the basis 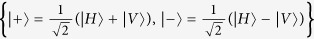, respectively. According to the outcomes of the detection, one performs corresponding operations (see Table 1) on atom *a*, which makes the two nonlocal atoms *a* and *b* collapse into the maximally entangled state


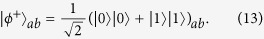


Our scheme for entanglement creation between two nonlocal atoms has some advantages. First, since the entangled photon pair produced by the PDC source emits from the middle point between the neighboring nodes (Alice and Bob) in the quantum repeater, the distance for quantum communication could be twice as much as that in the schemes using an ideal single-photon source. This releases the severe requirement of long coherence time for stationary qubits in realistic quantum communication. Second, the faulty scattering events between photons and two atoms can be heralded by the single-photon detectors. That is, if none of the detectors clicks in Alice (Bob), the nonlocal entanglement creation fails, which can be immediately discarded. Third, an arbitrary qubit error caused by the long noisy channels can be perfectly settled. In other words, the success probability of entanglement creation is free from the values of the collective noise parameters *γ*, *δ*, *ξ* and *λ*.

### Entanglement swapping of atomic systems assisted by a PDC source

**Figure 3:**
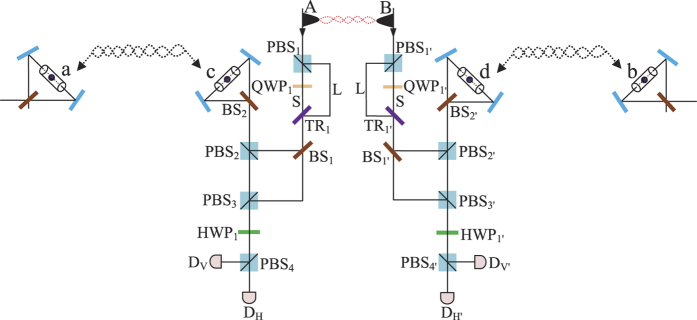
Schematic diagram for implementing entanglement swapping. *HWP*_*i*_ (*i* = 1, 1′) is a half-wave plate to complete a Hadamard operation (*H*_*p*_) on the polarization photon.

In a quantum repeater, one can extend the length of the quantum channel by local entanglement swapping^49^,^50^,^51^,^52^,^53^,^54^. The schematic diagram for our entanglement swapping protocol is shown in Fig. 3. Here, we consider two pairs of nonlocal atoms *ac* and *db*, which are both initially prepared in the maximally entangled states 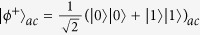 and 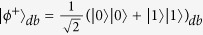, respectively. By performing a Bell-state measurement on local atoms *c* and *d*, we make the two nonlocal atoms *ab* collapse into the maximally entangled state 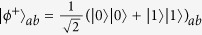, which indicates that a longer quantum channel is constructed. The principle of our entanglement swapping can be described as follows.

Suppose that an entangled photon pair AB produced by a PDC source is in the state 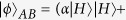

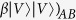
, where 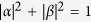. The initial state of the whole system is |Ψ_0_〉, where


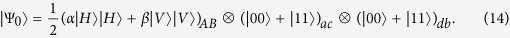


First, the photon A (B) passes through *PBS*_1_ (*PBS*_1′_) which transmits the photon component in state |*H*〉 and reflects the photon component in state |*V*〉. Owing to the fact that the interaction between photon A and atom *c* is identical to that between photon B and atom *d*, for simplicity, we just discuss the former part, and actually the latter part accomplishes the same process simultaneously. For photon A, the part in state |*H*〉 transmits through *PBS*_1_, *QWP*_1_, and *TR*_1_ via the short path (S), while the part in state |*V*〉 is reflected by *PBS*_1_ and *TR*_1_ via the long path (L). Since the two parts have the same processes, we only describe the interaction of the photon in the short path (S) in the following section. Then, the part in the short path (S) travels through a 50:50 beam splitter (*BS*_1_). The reflected component of this part is reflected by *PBS*_2_ into the scattering setup containing atom *c*, and travels through *PBS*_2_ and *PBS*_3_, while the transmitted component goes into *PBS*_3_ directly. The two parts of photon A are rejoined in *PBS*_3_. The same processes occur to the part in the long path (L) in a late time. After the nonlinear interaction, the state of the whole system is changed from |Ψ_0_〉 to |Ψ_1_〉. Here


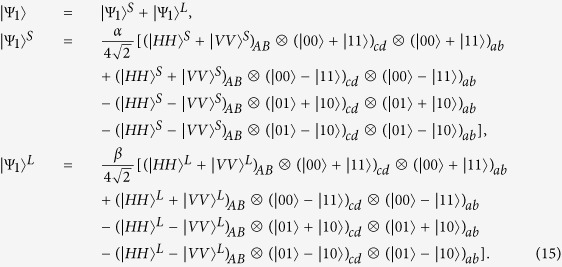


Second, a Hadamard operation *H*_*a*_ (e.g., using a *π*/2 microwave pulse or optical pulse^55^,^56^) is performed on local atoms *c* and *d* in the waveguides, respectively. Subsequently, by passing through *HWP*_1_ (*HWP*_1′_), the photon A (B) also gains a Hadamard operation *H*_*p*_. After that, the state of the whole system becomes


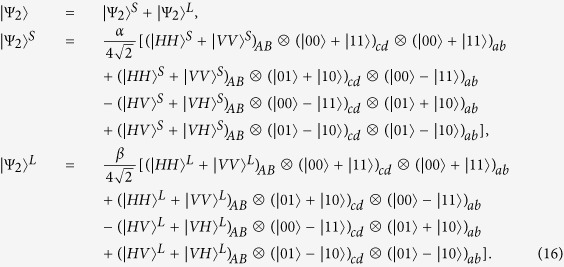


The photon A (B) travels through *PBS*_4_ (*PBS*_4′_) and is detected by single-photon detectors. Meanwhile, the state of atom *c* (*d*) is measured by external classical field.

Third, according to the outcomes of the photon detectors and the measurement of atom *c* (*d*), one can perform corresponding operations (see Table 2) on atom *a* to complete the entanglement swapping. Finally, after the processes mentioned above, the state of atoms *a* and *b* collapses into the maximally entangled state


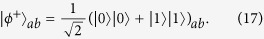


As the same as our entanglement creation scheme, in the quantum entanglement swapping protocol, the faulty scattering process between photons and atoms can also be heralded by single-photon detectors *D*_*H*_ (*D*_*H*′_) and *D*_*V*_ (*D*_*V*′_). Owing to the heralded mechanism, the overall success probability of our protocol may not be high, but the fidelity is 100%. Moreover, we make use of a usual PDC source to implement quantum swapping, which is easily available with compact setups in laboratory.

### Entanglement purification of atomic systems with PDC sources

**Figure 4:**
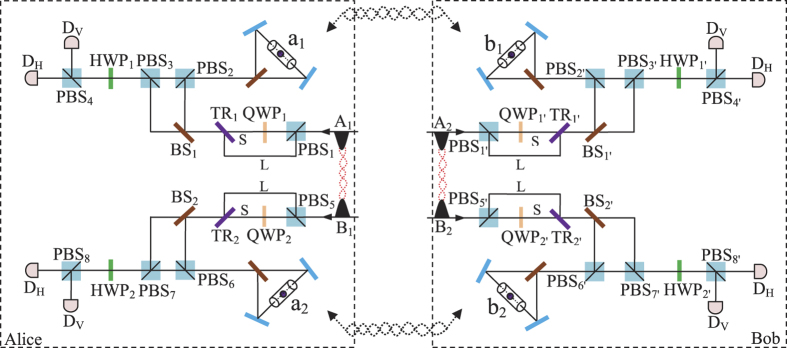
Schematic diagram showing the principle of atomic entanglement purification.

As mentioned above, we just care about the influence of noise on auxiliary photons in long quantum channels. However, the atomic qubits confined in 1D waveguides also inevitably suffer from noises, such as thermal fluctuation and the imperfection of the waveguides. In fact, utilizing entanglement concentration^57^,^58^,^59^, one can distill a subset system in a maximally entangled state from less-entangled pure state systems, and using entanglement purification^60^,^61^,^62^,^63^,^64^,^65^,^66^,^67^,^68^,^69^,^70^,^71^,^72^,^73^,^74^, one can obtain some maximally entangled states from a mixed state ensemble. Now, we start to explain our atomic entanglement purification protocol for bit-flip errors using the scattering of photons off single atoms in 1D waveguides, and its principle is shown in Fig. 4.

Suppose that the initial mixed state between atomic qubits *a* and *b*, owned by two remote parties Alice and Bob, respectively, can be written as


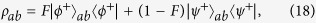


where 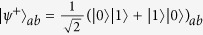 and *F* is the initial fidelity of the state |*ϕ*^+^〉. The two parties prepare two pairs of nonlocal entangled atoms: one is the source pair *a*_1_*b*_1_ and the other one is the target pair *a*_2_*b*_2_. When they select two pairs of entangled two-atom systems randomly, the four atoms are in the state 
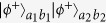
 with a probability of *F*^2^, 
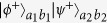
 and 
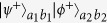
 with an equivalent probability of *F*(1 − *F*), and 
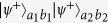
 with a probability of (1 − *F*)^2^, respectively. Our atomic entanglement purification protocol works with the following steps.

First, Alice and Bob prepare an entangled photon pair in the state 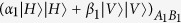 and 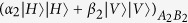 with PDC sources, respectively, and input them into the corresponding entanglement purification protocol. Here, 
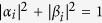
 (*i* = 1, 2). Owing to the fact that the process in Alice is the same as that in Bob, to simplify the discussion, we only describe the process in Alice. For Alice, the |*H*〉 and |*V*〉 components of photon *A*_1_ (*B*_1_) are spatially split by *PBS*_1_ (*PBS*_5_). Actually, the interaction between photon *A*_1_ and atom *a*_1_ is identical to that between photon *B*_1_ and atom *a*_2_, therefore we just discuss the process of the former part. In detail, the |*H*〉 component of photon *A*_1_ travels through *PBS*_1_, *QWP*_1_, and *TR*_1_ via the short path (S), while the |*V*〉 component is reflected by *PBS*_1_ and *TR*_1_ via the long path (L). Since the two parts have the same processes, we only talk about the interaction of the part in the short path (S) in the following section. Then, the part in the short path (S) goes through a 50:50 beam splitter (*BS*_1_). The reflected component of this part is reflected by *PBS*_2_ into the scattering setup containing atom *a*_1_, and goes through *PBS*_2_ and *PBS*_3_, while the transmitted component travels into *PBS*_3_ directly. The reflected and transmitted components are rejoined in *PBS*_3_. The identical process occurs to the part of photon A in the long path (L) in a late time.

Second, photon *A*_1_ travels through *HWP*_1_ and *PBS*_4_ and is probed by single-photon detectors. The same process occurs to photons *B*_1_, *A*_2_, and *B*_2_ simultaneously. There exist two kinds of measurement results. In detail, if two pairs of nonlocal entangled two-atom systems are initially in the state 
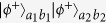
, the evolution of the whole system is


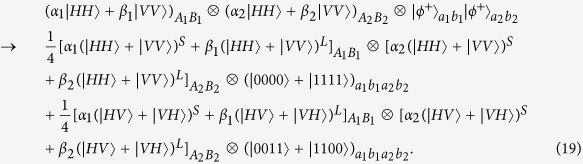


From Eq. (19), one can conclude that if the polarization measurements of photons *A*_1_ and *B*_1_ are the same (different) ones, the detections of *A*_2_ and *B*_2_ are also same (different), i.e., the result of the photon detection in Alice is consistent with that in Bob.

Similarly, the evolution of the other three cases can be described by:


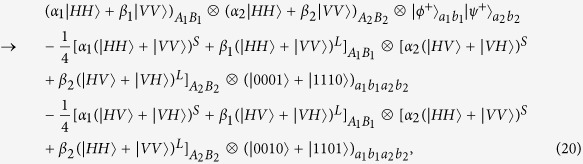



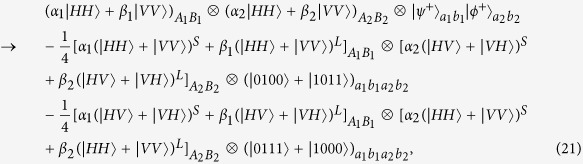


and


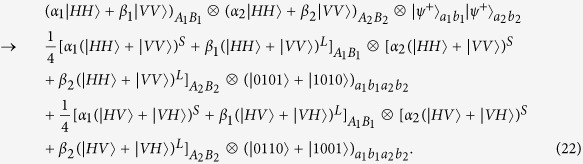


The measurements of photon polarization in four cases mentioned above are shown in Table 3.

Third, with the outcomes of the photon detection, the two parties can distill the two cases 
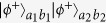
 and 
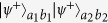
 with the probabilities of *F*^2^ and (1 − *F*)^2^, respectively. That is, based on our entanglement purification protocol, Alice and Bob can eventually preserve a new mixed state with a fidelity 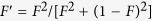, which is larger than *F* when 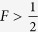. To recover the entangled state of atoms *a*_1_ and *b*_1_, they need perform a Hadamard operation *H*_*a*_ on atoms *a*_2_ and *b*_2_, respectively. Alice and Bob detect the states of atoms *a*_2_ and *b*_2_, and compare their results with classical communication. If the results are same, nothing needs to be done; otherwise, a *σ*_*z*_ operation needs to be put on atom *a*_1_.

In our entanglement purification protocol, the faulty events between emitters and photons can be heralded by the single-photon detectors, and that just decreases the efficiency of our protocols, not the fidelity. In other words, the entanglement purification protocol either succeeds with perfect fidelity or fails in a heralded way. As shown in Table 3, we adopt coincidence detection to complete the entanglement purification scheme. During the whole processes mentioned above, the photon scatters with the emitter in 1D waveguides only once, which reduces the probability of the faulty event’s occurrence as far as possible.

## Discussion

The efficiency of a quantum repeater is an important factor that should be considered for realistic long-distance quantum communication. In the following section, we will discuss this property of our protocols. Assuming that all the linear optical elements in our setups are perfect, the scattering process between photons and atoms in 1D waveguides becomes the key role that influences the performance of our scheme. To this end, we introduce the quantity 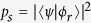 to describe the success probability of the scattering event in 1D waveguides. Here |*ψ*〉 and |*ϕ*_*r*_〉 are the spatial wave functions of the input photon and reflected photon component after the scattering process, respectively.

**Figure 5:**
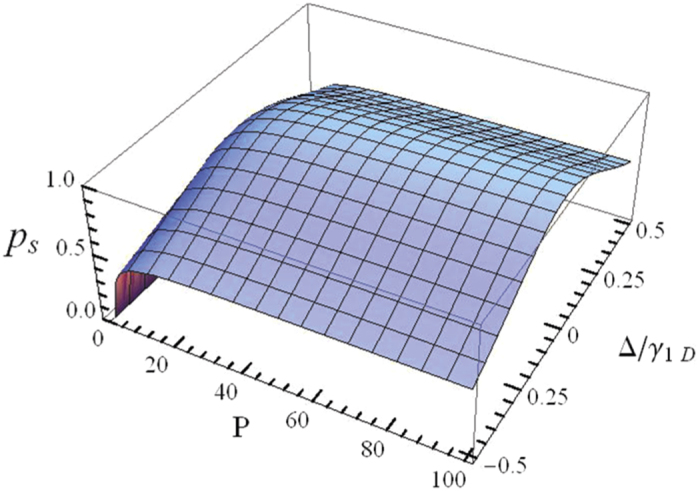
The success probability *p*_*s*_ of the scattering process vs the Purcell factor *P* and the detuning parameter Δ/*γ*_1*D*_.

**Figure 6:**
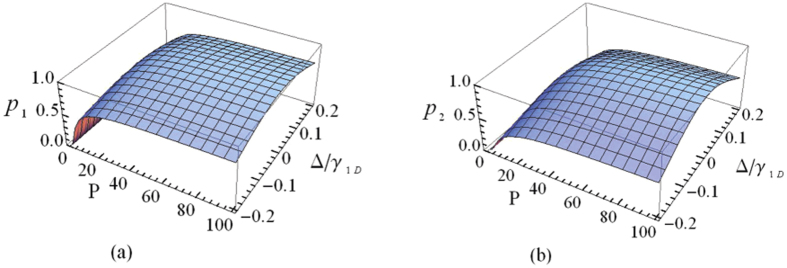
The success probabilities of our protocols vs the Purcell factor *P* and the detuning parameter Δ/*γ*_1*D*_. (**a**) The success probability *p*_1_ of our protocol for entanglement creation (entanglement swapping). (**b**) The success probability *p*_2_ of our protocol for entanglement purification.

As discussed above, the reflection coefficient for an incident photon scattering with the atom in a 1D waveguide is 
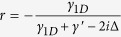
. With finite Purcell factor *P* and nonzero photonic detuning Δ, one easily gets the reflection coefficient *r* ≠ −1, that is, 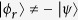. Figure 5 shows the success probability *p*_*s*_ of scattering event as a function of the Purcell factor *P* and the detuning parameter 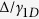. The success probability *p*_*s*_ can reach 80.0% when *P* = 10 and 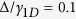, and 94.3% when *P* = 100 and Δ/*γ*_1*D*_ = 0.1, which indicates the positive role of the Purcell factor in determining the value of *p*_*s*_. In realistic systems^75^, a high Purcell factor *P* has been reported, and the photonic detuning can be well controlled. It is not difficult for us to obtain large reflection coefficient. To show the performance of our scheme for the heralded quantum repeater, we plot the success probabilities of our protocols as a function of Purcell factor *P* and detuning parameter Δ*/γ*_1*D*_, as illustrated in Fig. 6. Note that, *p*_1_ is defined as the success probability of entanglement creation or, equally, entanglement swapping, and *p*_2_ is the success probability of entanglement purification. As shown in Fig. 6, we find that for a given value of *P* (*P* = 20), with our scheme one achieves *p*_1_ = 82.3% and *p*_2_ = 67.7% as Δ*/γ*_1*D*_ = 0. While in the case *P* = 100 and Δ*/γ*_1*D*_ = 0, the success probabilities of our protocols are *p*_1_ = 96.1% and *p*_2_ = 92.3%, respectively. If the Purcell factor is *P* = 100, with Δ*/γ*_1*D*_ = 0.1, the corresponding success probabilities become *p*_1_ = 89.0% and *p*_2_ = 79.2%, respectively. The above observation is agreed with the prediction that the success probabilities of our protocols for the heralded quantum repeater will approach to 100% when *P* → ∞ and Δ*/γ*_1*D*_ = 0.

Note that, in practical situation, the polarization of output photon is swapped but 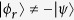 in Eq. (5), which causes a problem that the spatial wave functions in two arms of the interferometer no longer coincide. To solve the problem, we adopt a waveform corrector (*WFC*) in one arm of the interferometer. Actually, for successful events of imperfect processes, the waveform is 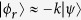 with |*k*| < 1. If the photon-atom detuning Δ is zero, 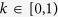, and the *WFC* can be realized by a beam splitter with the transmissivity *k*. When the photon-atom detuning Δ ≠ 0, the *WFC* may also consist of a delay to make the wave packets in two arms arrive at one place simultaneously.

Our scheme for the heralded quantum repeater based on atom-waveguide systems is particularly interesting because of its following characters. First, in our protocols, we make use of PDC sources to implement quantum communication. Nowdays, PDC sources are available with the current experimental technology, and have been widely used in various situations where entangled photon pairs are needed. Utilizing PDC sources, we make it possible to double the distance between two repeater nodes without influence of the noise coming from the increased quantum channels. Second, our scheme can turn the judgment of faulty events into the detection of the output photon polarization, which makes the fidelity of quantum repeater 100% in principle. In other words, the error-heralding mechanism ensures that our protocols either succeed with perfect fidelity or fail in a heralded way. As we know, if the entangled pairs are faulty, the fidelity of a realistic quantum repeater will decrease exponentially with the distance. Third, our scheme is also feasible in artificial solid-state systems, such as quantum dots embedded in a nanowire, superconducting quantum circuit coupled to transmission lines, and nitrogen-vacancy centers coupled to photonic-crystal waveguides. As mentioned above, our scheme is suitable for implementing realistic long-distance quantum communication. It is worth noting that the core of our scheme is the atom-waveguide system, in which a high Purcell factor has been obtained in experiment. With the great progress in the emitter-waveguide system^75^,^76^, there is no major technical obstacle to realize our scheme.

In summary, we have proposed a scheme for a heralded quantum repeater with the *Z* gate based on the scattering of photons off a four-level atom in 1D waveguides. In our protocols, we choose PDC sources to double the distance between two repeater nodes without increasing the negative influence of the collective noise in the channel. Moreover, the faulty scattering events can be abandoned by detecting the polarization of output photons, which ensures the fidelity to be 100% in principle. Benefiting from the great progress in controlling atom-waveguide systems, the atomic *Z* gate, i.e., the main component of our protocols, has been demonstrated. Therefore, our scheme for heralded quantum repeaters is feasible with current experimental technology. One may draw inspiration from our scheme in developing a new quantum repeater with a solid-state quantum system, such as quantum dots^77^,^78^,^79^,^80^ or nitrogen-vacancy centers^81^,^82^. Our repeater scheme will be useful in long-distance quantum communication in the future.

## Methods

### Single-photon dynamics

Owing to the fact that we only care about the interactions of near-resonant photons with the emitter, the quantum fields containing right- and left-going photons are completely separable^28^,^40^. Under this approximation, we can replace *a*_*k*_ in Eq. (1) with (*a*_*R*,*k*_ + *a*_*L*,*k*_). To obtain the transport property of the photon scattering with the emitter in a 1D waveguide, we assume that the photon initially comes from left with energy *E*_*k*_. The general wave function for the atom-photon system can be described by


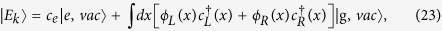


where *x* is the spatial coordinate along the waveguide, taking the origin *x* = 0 at the position of the atom, with positive to the right and negative to the left. 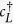 (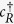) is a bosonic operator creating a left-propagating (right-propagating) photon, and 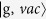 is the ground state of the system, meaning that there is no photon in the field and the atom is unexcited. The amplitudes of the photon wave-packets 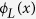 and 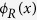 could be written as^28^


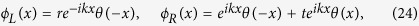


where *θ*(*x*) is Heaviside step function, *r* and *t* are the reflection and transmission coefficients, respectively. By solving the time-independent Schrödinger equation 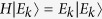, one can obtain the transmission coefficient *r* in Eq. (2).

### Realization of strong coupling between emitters and 1D waveguides

The atomic *Z* gate is an indispensable element in our scheme, which is based on the coupling between the emitters and 1D waveguides. In the past decade, great progress has been made to realize this strong coherent coupling in both theory and experiment. In 2005, Vlasov *et al*.^83^ reported that a Purcell factor *P* = 60 can be experimentally observed in low-loss silicon photonic crystal waveguides. In 2006, Chang *et al*.^84^ proposed a technique that realizes a dipole emitter coupled to a nanowire or a metallic nanotip, in which the Purcell factor reaches 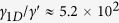 for a silver nanowire in principle. Subsequently, some similar schemes^78^,^85^ were demonstrated experimentally that a single optical plasmon in metallic nanowires is coupled to quantum dots. In 2008, Hansen *et al*.^79^ experimentally demonstrated that spontaneous emission from single quantum dots can be coupled very efficiently to a photonic crystal waveguide, where the emitter acts as a highly reflective mirror. In 2010, by coupling single InAs/GaAs semiconductor quantum dots to a photonic crystal waveguide mode, Thyrrestrup *et al*.^80^ measured a Purcell factor of *P* = 5.2 in experiment. In 2013, a Purcell factor of up to 8.3 was experimentally obtained by Kumar *et al*.^82^ with a propagating plasmonic gap mode residing in between two parallel silver nanowires. Meanwhile, Hung *et al*.^36^ put forward a scheme based on strong atom-photon interactions in 1D photonic crystal waveguides. In their proposal, one atom trapped in single nanobeam structure could provide a resonant probe with transmission 
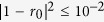
 in theory. In 2014, Goban *et al*.^86^ realized this scheme in experiment. Recently, Kolchin *et al*.^76^ presented a scheme in which a single emitter is coupled to a dielectric slot waveguide, and a high Purcell factor *P* = 31 is experimentally obtained.

## Additional Information

**How to cite this article**: Song, G.-Z. *et al*. Heralded quantum repeater based on the scattering of photons off single emitters using parametric down-conversion source. *Sci. Rep.* **6**, 28744; doi: 10.1038/srep28744 (2016).

## Figures and Tables

**Table 1:** The operations on atom *a* corresponding to the outcomes of the photon detectors in Alice and Bob.

**(Click in Alice)**	**(Click in Bob)**	**(Operation on atom** ***a***)
*D*_+_	*D*_+_	*I*
*D*_−_	*D*_−_	*I*
*D*_+_	*D*_−_	*σ*_*x*_
*D*_−_	*D*_+_	*σ*_*x*_

**Table 2:** The operations on atom *a* corresponding to the results of the photon detectors and the states of atoms *c* and *d*.

**Detections of photons A and B**	**States of atoms** ***c*** **and** ***d***	**Operation on atom** ***a***
same	same	*I*
same	different	*σ*_*z*_
different	same	*σ*_*x*_
different	different	*σ*_*x*_*σ*_*z*_

Note that, for the measurement of photons A and B, the “same” event includes two cases: *D*_*H*_*D*_*H*′_ and *D*_*V*_*D*_*V*′_; the “different” event includes the other two cases: *D*_*H*_*D*_*V*′_ and *D*_*V*_*D*_*H*′_.

**Table 3:** The measurement results of photon pairs *A*_1_ *B*_1_ and *A*_2_ *B*_2_ corresponding to the initial entangled states of the four atoms.

**Initial**	**states**	**Photon**	**measurement**
(***a***_**1**_***b***_**1**_)	(***a***_**2**_***b***_**2**_)	***A***_**1**_ **and** ***B***_**1**_	***A***_**2**_ **and** ***B***_**2**_
|*ϕ*^+^〉	|*ϕ*^+^〉	same (different)	same (different)
|*ϕ*^+^〉	|*ψ*^+^〉	same (different)	different (same)
|*ψ*^+^〉	|*ϕ*^+^〉	same (different)	different (same)
|*ψ*^+^〉	|*ψ*^+^〉	same (different)	same (different)
